# Polymorphism of Promoter Region of TNFRSF1A Gene (−610 T > G) as a Novel Predictive Factor for Radiotherapy Induced Oral Mucositis in HNC Patients

**DOI:** 10.1007/s12253-017-0227-1

**Published:** 2017-04-11

**Authors:** Anna Brzozowska, Tomasz Powrózek, Iwona Homa-Mlak, Radosław Mlak, Marzanna Ciesielka, Paweł Gołębiowski, Teresa Małecka-Massalska

**Affiliations:** 1 Department of Oncology, Medical University of Lublin, Jaczewskiego 7, 20-090 Lublin, Poland; 2 Department of Human Physiology, Medical University of Lublin, Radziwiłłowska 11, 20-080 Lublin, Poland; 3 Department of Forensic Medicine, Medical University of Lublin, Jaczewskiego 8, 20-090 Lublin, Poland

**Keywords:** Oral mucositis, Radiotherapy, Head and neck cancer, Polymorphism, TNFRSF1A

## Abstract

Every year, about 650 thousand new cases of Head and Neck Cancer (HNC) are diagnosed globally. Apart from surgery, radiotherapy (RTH), chemotherapy (CHT) or its combination is used in the treatment of HNC. One of the most frequent complications and, at the same time, limitations of RTH is oral mucositis (OM). Proinflammatory cytokines (including TNF-α) play a key role in the development of OM. Genetic alterations, i.e. single nucleotide polymorphisms (SNPs) within genes encoding for receptors for TNF (ie. TNFRSF1A) may change their function. The aim of this study was to investigate relationship between a polymorphism of TNFRSF1A and occurrence and severity of acute reaction after RTH for HNC patients. Data from 58 HNC patients (stages I-IV) were analyzed. All of them were irradiated using IMRT technique with doses 50-70Gy. Oral mucositis (OM) was evaluated according to RTOG/EORTC guidelines. DNA from HNC patients were isolated from whole blood and genotypes were determined by sequencing method. Patients with TT or GT genotype demonstrated higher risk of manifestation of grade 3 OM in 5th week of RTH (p=0.041; OR=9.240; 95% CI: 1.101–77.581) compared to GG carriers. Similarly, high risk of grade 3 OM in patients with T allele presence was noted in 6th week (p=0.030; OR=10.50; 95%CI:1.257–87.690) and in 7th week (p=0.008; OR=5.625; 95% CI: 1.584–19.975) of treatment compared to patients with GG homozygote. Our results indicate an association between SNP of TNFRSF1A (rs4149570) gene and risk of more severe OM related to radiation therapy for HNC patients.

## Introduction

Every year, about 650 thousand new cases of Head and Neck Cancer (HNC), located in the area of oral cavity, pharynx, larynx, sinuses and salivary glands, are diagnosed globally. HNC is the sixth most prevalent neoplasm accounting for about 6% of all cancerous lesions [1].

Apart from surgery, radiotherapy (RTH) often combined with chemotherapy plays a crucial role in the treatment of HNC. One of the most frequent complications and at the same time limitations of RTH is acute oral mucositis (OM) occurring on almost 100% of HNC patients especially when using unconventional radiation schemes and combining radio- chemotherapy (RCTH) [2]. OM is observed also in 40% of patients subjected to chemotherapy alone, especially when using 5-fluorouracil, methotrexate, doxorubicin, taxanes or purine antagonists [3]. It is of special significance with the current trend of frequently combining radiotherapy and chemotherapy in HNC patients.

OM is characterized with gradually increasing edema of mucous membranes, oral erythema, ulcerations, pain and dysphagia. In 34% patients there occurs severe OM (of 3rd and 4th degree in RTOG/EORTC -Radiation Therapy Oncology Group /European Organisation for Research and Treatment of Cancer, scale) [2, 4]. Severe OM leads to worsening of the quality of patients’ life, hospitalizations and interruptions of radiotherapy [2]. In 30–35% of HNC cases with severe OM, it is necessary to delay or discontinue further courses of chemotherapy and in 60%, to reduce the doses of chemotherapeutics [5]. Limitations in the application of the complete treatment scheme influence the results of local disease control and overall survival because each 5 days’ interruption in RTH increases the risk of progression by 14% [6].

Despite the known OM risk factors such as old age and male gender, oral hygiene, high dose of radiation, smoking, systemic diseases, RTH technique and combined RCTH, so far no factors have been identified which would facilitate precise estimation of the risk of occurrence and intensity of OM [7]. However, the observed high individual variability in the development of OM indicates significant role of genetic predispositions [8–10].

Among many mechanisms taking part in the development of OM caused by ionizing radiation and/or chemotherapy, the decisive role is played by proinflammatory cytokines including tumor necrosis factor-alfa (TNF-α), responsible for the regulation of two opposite processes: proliferation and apoptosis [11]. The apoptotic pathway is activated by TNF through TNF1 receptor (TNFR1) and it cannot be ruled out that the disturbances of apoptosis in OM are indeed caused by the dysfunction of this receptor [12]. Genetic alterations such as single nucleotide polymorphisms (SNPs) located in the promoter regions of genes encoding for the TNF receptors (e.g. TNFRSF1A) may influence the level of their expression and function. It may potentially modulate the risk of occurrence and intensity of OM in HNC patients treated with RTH.

Several trials have been conducted so far confirming a potential association between the genetic alterations (SNPs, mutations, expression and other) in the genes encoding proteins (ligands or their receptors) which are taking part in inflammatory processes and the occurrence of OM in HNC patients treated with RTH [8–11].

Thus, the aim of this study was the evaluation of the relationship between SNP (rs4149570, c.-1187 T > G) of TNFRSF1A and the occurrence and intensity of OM in HNC patients which were treated with RTH.

## Materials and Methods

### Patient and Clinical Data

We recruited 58 patients with advanced HNC (stage I-IV). They were diagnosed and treated between 2014 and 2015 at the Oncology Department Medical University in Lublin. We collected detailed information about demographic and clinical data (Table 1). The stage of the disease was evaluated on the basis of the TNM classification (VII edition by UICC). Alcohol consumption was evaluated according to the International Statistical Classification of Diseases and Related Health Problems (ICD) as excessive (F10.1 and F 10.2) or occasional.

**Table 1:** Characteristics of the study group

Factor		Study group (*n* = 58)
Gender	Male	47 (81%)
	Female	11 (19%)
Age (years)	Median ± SD (standard deviation)	60 ± 11
	≥ 60	36 (62.1%)
	< 60	22 (37.9%)
Performance status (PS)	1	54 (93.1%)
	2	4 (6.9%)
Histopathological diagnosis	Squamous cell carcinoma	52 (89.7%)
	Others	6 (10.3%)
Disease stage	I	2 (3.4%)
	III	11 (19%)
	IVA	40 (69%)
	IVB	5 (8.6%)
Tumor location	Oral cavity	3 (5.2)
	Oropharynx	7 (12.1)
	Hypopharynx	6 (10.3)
	Larynx	34 (58.6)
	Other (sinus)	8 (13.8)
Neoadjuvant chemotherapy	Yes	9 (15.5%)
	No	49 (84.5%)
Prior surgical treatment	Yes	43 (74.1%)
	No	15 (25.9%)
Concurrent chemotherapy (RTCH)	Yes	21 (36.2%)
	No	37 (63.8%)
Comorbidity	Yes	37 (63.8%)
	No	21 (36.2%)
Smoking status	Ever	49 (84.5%)
	Never	9 (15.5%)
Alcohol consumption	Excessive	26 (44.8%)
	Occasional	32 (55.2%)

This project was approved by Bioethical Commission in Medical University in Lublin (KE-0254/232/2014). Patients were informed about the purpose of the study and they signed a consent for this research.

### Radiotherapy

Patients were treated with radical radiotherapy using linear accelerator ONCOR (Siemens). Using IMRT technique total doses of 66–70 Gy (daily dose 2 Gy) were prescribed. Patients with gross disease were treated with total dose 70 Gy in 35 fractions for tumor and enlarged lymph nodes. Elective lymph nodes were treated with doses of 54 Gy or 60 Gy; Patients after surgical resection were given a dose of 66 Gy in 33 fractions for high risk volume, the intermediate and low risk subclinical volumes received 60 Gy and 54 Gy, respectively.

Moreover patients were treated with chemotherapy based on cisplatine and 5-fluorouracil (PF) schemes. 1 to 4 courses of chemotherapy were administered.

### Genotyping

**Fig. 1:**
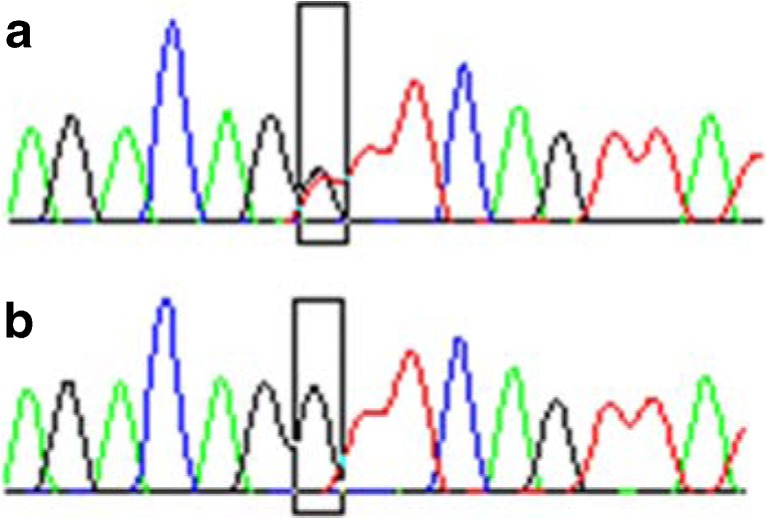
An example of results of TNF receptor gene sequencing. Changes in the SNP (rs414141) were marked with a *black box*. Figure 1 **a** and **b** shows the GT heterozygous and GG homozygous patients respectively

We obtained peripheral blood from all patients and isolated the DNA [using DNA Blood Mini Kit (Qiagen, Canada)]. Subsequently we measured DNA concentration using NanoDrop Lite Spectrophotometer (Thermo Fisher Scientific, USA)]. SNP genotypes were determined using the sequencing technique (3500 Genetic Analyzer, Life Technologies). For the reaction a kit of BigDye® Terminator v3.1 Sequencing Standard Kit (Thermo Fisher Scientific, USA) was used. Fig. 1 shows an example of the result of sequencing.

### Toxicity Assessment

The evaluation of reaction after radiation was performed before treatment and on every week of radiotherapy using RTOG/EORTC scale.

### Statistical Analysis

Genotyping result of studied SNP was retrospectively correlated with clinical and demographic factors as well as severity of patients’ radiation-induced skin reactions. Statistical analysis of the results was conducted using MedCalc version 12 (MedCalc software, Belgium) computer software. Using Chi-square test, the balance of Hardy-Weinberg equilibrium was assessed. Fisher’s exact test was used to compare genotypes frequency with clinicopathologic factors and presence of skin toxicity in head and neck cancer patients. Odds ratio (OR) was calculated to assess the risk of radiation-induced skin reaction development among genotypes carriers. *p* values <0.05 were considered as statistically significant.

## Results

Based on the conducted direct sequencing analysis the following genotypes distribution of TNFRSF1A (rs4149570, c.-1187 T > G) was determined: TT in 5 (8.6% of studied group), GT in 31 (53.5%) and GG in 22 patients (37.9%), respectively. Distribution of TNFRSF1A genotypes was in Hardy-Weinberg (*p* = 0.197; χ2 = 1.666) equilibrium and was not dependent on patients’ clinical and demographic factors (Table 2).

**Table 2:** *TNFRSF1A* genotype distribution according to patients’ clinical and demographic factors

*TNFRSF1A* rs4149621					
Factor		TT (*n* = 5; 8.6%)	GG (*n* = 22; 37.9%)	GT (*n* = 31; 53.5%)	*p*
Gender	Male	4 (8.5%)	21(44.7%)	22 (46.8%)	0.148
	Female	1 (9.1%)	1 (9.1%)	9 (81.8%)	
Age (years)	≥ 60	1 (2.8%)	17 (47.2%)	18 (50%)	0.148
	< 60	4 (18.2%)	5 (22.7%)	13 (59.1%)	
Performance status (PS)	1	5 (9.3%)	20 (37%)	29 (53.7%)	0.932
	2	0	2 (50%)	2 (50%)	
Histopathological diagnosis	Squamous cell carcinoma	5 (9.6%)	20 (38.5%)	27 (51.9%)	0.973
	Others	0	2 (33.3)	4 (66.7%)	
Disease stage	I and III	1 (7.7%)	4 (30.8%)	8 (61.5%)	0.874
	IV	4 (8.9%)	18 (40%)	23 (51.1%)	
Tumor location	Oropharynx, Hypopharynx, Larynx,	5 (10)	21 (42)	24 (48)	0.1111
	Oral cavity, Sinus,	0	1 (12.5)	7 (87.5)	
	Larynx	5 (14.7%)	14 (41.2%)	15 (44.1%)	0.218
	Others	0	8 (33.3%)	16 (66.7%)	
Alcohol consumption	Excessive	1 (3.9%)	9 (34.6%)	16 (61.5%)	0.669
	Occasional	4 (12.5%)	13 (40.6%)	15 (46.9%)	
Concurrent CTH	Yes	4 (9.1%)	7 (33.3%)	10 (47.6%)	0.274
	No	1 (2.7%)	15 (40.5%)	21 (56.8%)	

During RTH treatment every patient enrolled to this study developed radiation-induced skin and mucosa reactions (grade 0–3 according to RTOG). Intensification of RTH side effects was observed each week of radiation. After first week of the treatment patients demonstrated lack or only mild toxicity of applied therapy (grade 0 in 10.3% of patients and grade 1 in 89.7% of patients). The severity of mucositis toxicity increased after subsequent weeks of RTH (after 3th week the grade 2–3 of acute mucositis reaction was observed in 65.5% of patients, whereas after 7th week in 91.4% of study participants) (Tables 3 and 4).

**Table 3:** Radiation reaction severity (oral mucositis) distribution according to patients’ clinical and demographic factors after subsequent cycles of RTH

Factor		RTH cycle and grade of radiation induced skin reaction								
		5th week of RTH Radiation reaction grade		*p*, OR [95%CI]	6th week of RTH Radiation reaction grade		*p*, OR [95%CI]	7th week of RTH Radiation reaction grade		*p*, OR [95%CI]
		1	2 and 3		1	2 and 3		1	2 and 3	
Gender	Male	6 (12.8%)	41 (87.2%)	0.394 3.602 [0.189–68.81]	6 (12.8%)	41 (87.2%)	0.394 3.602 [0.189–68.81]	5 (10.6%)	42 (89.4%)	0.471 2.977 [0.153–57.87]
	Female	0	11		0	11		0	11	
Age	≥60	6 (16.7%)	30 (83.3%)	0.130 9.590 [0.513–179.17]	6 (16.7%)	30 (83.3%)	0.130 9.590 [0.513–179.17]	5 (13.9%)	31 (86.1%)	0.170 7.857 [0.413–149.40]
	<60	0	22		0	22		0	22	
Disease stage	I and III	2 (15.4%)	11 (84.6%)	0.503 1.864 [0.301–11.539]	2 (15.4%)	11 (84.6%)	0.503 1.864 [0.301–11.539]	2 (15.4%)	11 (84.6%)	0.503 1.864 [0.301–11.539]
	IV	4 (8.9%)	41 (91.1%)		4 (8.9%)	41 (91.1%)		4 (8.9%)	41 (91.1%)	
Smoking history	Smoker	5 (10.2%)	44 (89.8%)	0.935 0.909 [0.093–8.847]	5 (10.2%)	44 (89.8%)	0.935 0.909 [0.093–8.847]	5 (10.2%)	44 (89.8%)	0.574 2.348 [0.119–46.171]
	Non-smoker	1 (11.1%)	8 (88.9%)		1 (11.1%)	8 (88.9%)		0	9	
Alcohol consumption	Excessive	2 (7.7%)	24 (92.3%)	0.554 0.583 [0.098–3.469]	2 (7.7%)	24 (92.3%)	0.554 0.583 [0.098–3.469]	1 (3.8%)	25 (96.2%)	0.269 0.280 [0.029–2.675]
	Occasional	4 (12.5%)	28 (87.5%)		4 (12.5%)	28 (87.5%)		4 (12.5%)	28 (87.5%)	
Concurent chemotherapy	Yes	0	21	0.144 0.113 [0.006–2.107]	0	21	0.144 0.113 [0.006–2.107]	0	21	0.144 0.113 [0.006–2.107]
	No	6 (16.2%)	31 (83.8%)		6 (16.2%)	31 (83.8%)		6 (16.2%)	31 (83.8%)	
Neoadjuvant chemotherapy	Yes	0	9	0.490 0.352 [0.018–6.803]	0	9	0.490 0.352 [0.018–6.803]	0	9	0.490 0.352 [0.018–6.803]
	No	6 (12.2%)	43 (87.8%)		6 (12.2%)	43 (87.8%)		6 (12.2%)	43 (87.8%)

**Table 4:** Radiation reaction severity (oral mucositis) distribution according to patients’ clinical and demographic factors after subsequent cycles of RTH

Factor		RTH cycle and grade of radiation induced skin reaction								
		5th week of RTH Radiation reaction grade		*p*, OR [95%CI]	6th week of RTH Radiation reaction grade		*p*, OR [95%CI]	7th week of RTH Radiation reaction grade		*p*, OR [95%CI]
		1 and 2	3		1 and 2	3		1 and 2	3	
Gender	Male	39 (83%)	8 (17%)	0.165 0.359 [0.084–1.522]	38 (80.9%)	9 (19.1%)	0.227 0.492 [0.099–1.727]	28 (59.6%)	19 (40.4%)	0.761 0.814 [0.217–3.055]
	Female	7 (63.6%)	4 (36.4%)		7 (63.6%)	4 (36.4%)		6 (54.5%)	5 (45.5%)	
Age	≥60	30 (83.3%)	6 (16.7%)	0.337 1.875 [0.519–6.771]	29 (80.6%)	7 (19.4%)	0.490 1.554 [0.445–5.421]	24 (66.7%)	12 (33.3%)	0.115 2.40 [0.808–7.126]
	<60	16 (72.7%)	6 (27.3%)		16 (72.7%)	6 (27.3%)		10 (45.5%)	12 (54.5%)	
Disease stage	I and III	11 (84.6%)	2 (15.4%)	0.594 1.571 [0.298–8.285]	9 (69.2%)	4 (30.8%)	0.416 0.563 [0.141–2.249]	8 (61.5%)	5 (38.5%)	0.809 1.169 [0.330–4.140]
	IV	35 (77.8%)	10 (22.2%)		36 (80%)	9 (20%)		26 (57.8%)	19 (42.2%)	
Smoking history	Smoker	40 (81.6%)	9 (18.4%)	0.317 2.222 [0.466–10.609]	39 (79.6%)	10 (20.4%)	0.399 1.950 [0.414–9.190]	30 (61.2%)	19 (38.8%)	0.353 1.974 [0.470–8.288]
	Non-smoker	6 (66.7%)	3 (33.3%)		6 (66.7%)	3 (33.3%)		4 (44.4%)	5 (55.6%)	
Alcohol consumption	Excessive	20 (76.9%)	6 (23.1%)	0.686 0.769 [0.215–2.747]	19 (73.1%)	7 (26.9%)	0.460 0.626 [0.181–2.166]	11 (42.3%)	15 (57.7%)	0.026 3.485 [1.166–10.418]
	Occasional	26 (81.3%)	6 (18.7%)		26 (81.3%)	6 (18.7%)		23 (71.9%)	9 (28.1%)	
Concurent chemotherapy	Yes	13 (61.9%)	8 (38.1%)	0.019 5.077 [1.302–19.802]	15 (71.4%)	6 (28.6%)	0.400 0.583 [0.166–2.045]	6 (28.6%)	15 (71.4%)	<0.001 7.778 [2.323–26.043]
	No	33 (89.2%)	4 (10.8%)		30 (81.1%)	7 (18.9%)		28 (75.7%)	9 (24.3%)	
Neoadjuvant chemotherapy	Yes	7 (77.8%)	2 (22.2%)	0.902 0.897 [0.161–5.003]	5 (55.6%)	4 (44.4%)	0.098 0.281 [0.063–1.261]	3 (33.3%)	6 (66.7%)	0.107 0.290 [0.065–1.305]
	No	39 (79.6%)	10 (20.4%)		40 (81.6%)	9 (18.4%)		31 (63.3%)	18 (36.7%)	

Among the known risk factors such as: gender, age, disease stage, smoking history, alcohol consumption, concurrent and neoadjuvant chemotherapy, only alcohol consumption and concurrent chemotherapy significantly increased the OM intensity. In the group of patients with excessive alcohol consumption, the 3rd degree of OM occurred significantly more frequently, compared to the 1st and 2nd degree, in the 7th week of RTH (*p* = 0.026; OR = 3.485; 95% CI:1.166–10.418). Among the patients with concurrent chemotherapy the 3rd degree of OM occurred significantly more frequently than the 1st and 2nd degree in the 5th and 7th week of RTH (*p* = 0.0195; OR = 077; 95% CI:1.302–19.802) and (*p* < 0.0017; OR = 778; 95% CI: 2.323–26.043), respectively.

The radiation-induced mucositis toxicity during subsequent weeks of RTH did not depend on patients’ genotype distribution (Table 5). However, patients with diagnosed more severe mucositis toxicity (grade 3) in 7th week of RTH carried TT genotype (*p* = 0.037), and milder side effects (grade 1 and 2) were observed more frequently in patients with presence of GG homozygote (*p* = 0.008) compared to T allele carriers. Based on the study results we found that studied SNP of TNFRSF1A had significant effect on the severity of acute radiation-induced mucositis toxicity. The presence of T allele in patients’ genotypes was associated with higher risk of development of more severe side effects of therapy in 5th, 6th and 7th week of RTH. Patients with TT or GT genotype demonstrated higher risk of manifestation of grade 3 mucositis toxicity in 5th week of RTH (*p* = 0.041; OR = 9.240; 95% CI: 1.101–77.581) compared to GG carriers in whose grade 1 and 2 of mucositis reactions was observed more frequently. The same high risk tendency to develop severe (grade 3) side effects of RTH in patients with T allele presence was noted in 6th week (*p* = 0.030; OR = 10.50; 95%CI:1.257–87.690) and in 7th week (*p* = 0.008; OR = 5.625; 95% CI: 1.584–19.975) of treatment compared to patients with GG homozygote. All results assessing impact of studied SNP on RTH toxicity are presented in Tables 5 and 6.

**Table 5:** *TNFRSF1A* genotype distribution according to patients’ radiation-induced skin reactions severity after subsequent cycles of RTH

RTH week	Radiation reaction grade	TT (*n* = 5; 8.6%)	GG (*n* = 22; 37.9%)	GT (*n* = 31; 53.5%)	*p*
1	0 (*n* = 6; 10.3%)	1 (16.7%)	3 (50%)	2 (33.3%)	0.905
	1 (*n* = 52; 89.7%)	4 (7.7%)	19 (36.5%)	29 (55.8%)	
2	1 (*n* = 34; 58.6%)	5 (14.7%)	13 (38.2%)	16 (47.1%)	0.297
	2 (*n* = 24; 41.4%)	0	9 (37.5%)	15 (62.5%)	
3	1 (*n* = 20; 34.5%)	2 (10%)	9 (45%)	9 (45%)	0.813
	2 and 3 (*n* = 38; 65.5%)	3 (7.9%)	13 (34.2%)	22 (57.9%)	
	1 and 2 (*n* = 54; 93.1%)	5 (9.3%)	20 (37%)	29 (53.7%)	0.932
	3 (*n* = 4; 6.9%)	0	2 (50%)	2 (50%)	
4	1 (*n* = 11; 19%)	1 (9.1%)	6 (54.5%)	4 (36.4%)	0.554
	2 and 3 (*n* = 47; 81%)	4 (8.5%)	16 (34%)	27 (57.4%)	
	1 and 2 (*n* = 47; 81%)	4 (8.5%)	20 (42.6%)	23 (48.9%)	0.440
	3 (*n* = 11; 19%)	1 (9.1%)	2 (18.2%)	8 (72.7%)	
5	1 (*n* = 6; 10.3%)	0	4 (66.7%)	2 (33.3%)	0.635
	2 and 3 (*n* = 52; 89.7%)	5 (9.6%)	18 (34.6%)	29 (55.8%)	
	1 and 2 (*n* = 46; 79.3%)	3 (6.5%)	21 (45.7%)	22 (47.8%)	0.157
	3 (*n* = 12; 20.7%)	2 (16.7%)	1 (8.3%)	9 (75%)	
6	1 (*n* = 6; 10.3%)	0	4 (66.7%)	2 (33.3%)	0.635
	2 and 3 (*n* = 52; 89.7%)	5 (9.6%)	18 (34.6%)	29 (55.8%)	
	1 and 2 (*n* = 45; 77.6%)	3 (6.6%)	21 (46.7%)	21 (46.7%)	0.108
	3 (*n* = 13; 22.4%)	2 (15.4%)	1 (7.7%)	10 (76.9%)	
7	1 (*n* = 5; 8.6%)	0	3 (60%)	2 (40%)	0.890
	2 and 3 (*n* = 53; 91.4%)	5 (9.4%)	19 (35.8%)	29 (54.7%)	
	1 and 2 (*n* = 34; 58.6%)	1 (2.9%)	18 (52.9%)	15 (44.1%)	0.037
	3 (*n* = 24; 41.4%)	4 (16.7%)	4 (16.7%)	16 (66.6%)

**Table 6:** Impact of *TNFRSF1A* genotypes on the risk of patients’ radiation-induced skin reactions severity after subsequent cycles of RTH

RTH week	Radiation reaction grade	TT (*n* = 5; 8.6%)	GG or GT	*p*, OR [95%CI]	GG (*n* = 22; 37.9%)	TT or GT	*p*, OR [95%CI]	GT (*n* = 31; 53.5%)	TT or GG	*p*, OR [95%CI]
1	0 (*n* = 6; 10.3%)	1 (16.7%)	5 (83.3%)	0.470 2.40 [0.222–25.855]	3 (50%)	3 (50%)	0.524 1.737 [0.318–9.479]	2 (33.3%)	4 (66.7%)	0.309 0.397 [0.067–2.360]
	1 (*n* = 52; 89.7%)	4 (7.7%)	48 (92.3%)		19 (36.5%)	33 (63.5%)		29 (55.8%)	23 (44.2%)	
2	1 (*n* = 34; 58.6%)	5 (14.7%)	29 (85.3%)	0.141 9.136 [0.481–173.546]	13 (38.2%)	21 (61.8%)	0.955 1.032 [0.351–3.031]	16 (47.1%)	18 (52.9%)	0.248 0.533 [0.184–1.549]
	2 (*n* = 24; 41.4%)	0	24 (100%)		9 (37.5%)	15 (62.5%)		15 (62.5%)	9 (37.5%)	
3	1 (*n* = 20; 34.5%)	2 (10%)	18 (90%)	0.786 1.296 [0.198–8.473]	9 (45%)	11 (55%)	0.422 1.573 [0.520–4.760]	9 (45%)	11 (55%)	0.351 0.595 [0.200–1.772]
	2 and 3 (*n* = 38; 65.5%)	3 (7.9%)	35 (82.1%)		13 (34.2%)	25 (65.8%)		22 (57.9%)	16 (42.1%)	
	1 and 2 (*n* = 54; 93.1%)	5 (9.3%)	49 (90.7%)	*ns*	20 (37%)	34 (63%)	0.610 0.588 [0.077–4.507]	29 (53.7%)	25 (46.3%)	0.886 1.160 [0.152–8.847]
	3 (*n* = 4; 6.9%)	0	4 (100%)		2 (50%)	2 (50%)		2 (50%)	2 (50%)	
4	1 (*n* = 11; 19%)	1 (9.1%)	10 (90.9%)	0.951 1.075 [0.108–10.689]	6 (54.5%)	5 (45.5%)	0.214 2.325 [0.614–8.802]	4 (36.4%)	7 (63.6%)	0.215 0.423 [0.109–1.646]
	2 and 3 (*n* = 47; 81%)	4 (8.5%)	43 (91.5%)		16 (34%)	31 (66%)		27 (57.4%)	20 (42.6%)	
	1 and 2 (*n* = 47; 81%)	4 (8.5%)	43 (91.5%)	0.951 0.930 [0.094–9.249]	20 (42.6%)	27 (57.4%)	0.150 3.333 [0.648–17.144]	23 (48.9%)	24 (51.1%)	0.165 0.359 [0.085–1.524]
	3 (*n* = 11; 19%)	1 (9.1%)	10 (90.9%)		2 (18.2%)	9 (81.8%)		8 (72.7%)	3 (27.3%)	
5	1 (*n* = 6; 10.3%)	0	6 (100%)	0.790 0.664 [0.033–13.463]	4 (66.7%)	2 (33.3%)	0.146 3.778 [0.630–22.649]	2 (33.3%)	4 (66.7%)	0.309 0.397 [0.067–2.360]
	2 and 3 (*n* = 52; 89.7%)	5 (9.6%)	47 (90.4%)		18 (34.6%)	34 (65.4%)		29 (55.8%)	23 (44.2%)	
	1 and 2 (*n* = 46; 79.3%)	3 (6.5%)	43 (93.5%)	0.282 0.349 [0.051–2.372]	21 (45.7%)	25 (54.3%)	0.041 9.240 [1.101–77.581]	22 (47.8%)	24 (52.2%)	0.104 0.306 [0.073–1.276]
	3 (*n* = 12; 20.7%)	2 (16.7%)	10 (83.3%)		1 (8.3%)	11 (91.7%)		9 (75%)	3 (25%)	
6	1 (*n* = 6; 10.3%)	0	6 (100%)	0.790 0.664 [0.033–13.463]	4 (66.7%)	2 (33.3%)	0.146 3.778 [0.630–22.649]	2 (33.3%)	4 (66.7%)	0.309 0.397 [0.067–2.360]
	2 and 3 (*n* = 52; 89.7%)	5 (9.6%)	47 (90.4%)		18 (34.6%)	34 (65.4%)		29 (55.8%)	23 (44.2%)	
	1 and 2 (*n* = 45; 77.6%)	3 (6.6%)	42 (93.4%)	0.337 0.393 [0.058–2.645]	21 (46.7%)	24 (53.3%)	0.030 10.50 [1.257–87.690]	21 (46.7%)	24 (53.3%)	0.064 0.263 [0.064–1.083]
	3 (*n* = 13; 22.4%)	2 (15.4%)	11 (84.6%)		1 (7.7%)	12 (92.3%)		10 (76.9%)	3 (23.1%)	
7	1 (*n* = 5; 8.6%)	0	5 (100%)	0.886 0.802 [0.039–16.533]	3 (60%)	2 (40%)	0.302 2.684 [0.412–17.507]	2 (40%)	3 (60%)	0.533 0.552 [0.085–3.577]
	2 and 3 (*n* = 53; 91.4%)	5 (9.4%)	48 (90.6%)		19 (35.8%)	34 (64.2%)		29 (54.7%)	24 (45.3%)	
	1 and 2 (*n* = 34; 58.6%)	1 (2.9%)	33 (97.1%)	0.102 0.152 [0.016–1.453]	18 (52.9%)	16 (47.1%)	0.008 5.625 [1.584–19.975]	15 (44.1%)	19 (55.9%)	0.093 0.395 [0.133–1.169]
	3 (*n* = 24; 41.4%)	4 (16.7%)	20 (83.3%)		4 (16.7%)	20 (83.3%)		16 (66.6%)	8 (33.4%)	

## Discussion

At least 14 different mechanisms and metabolic pathways take part in the development of OM in patients treated with combined radiochemotherapy due to HNC [13]. The best known mechanism is the generation and function of proinflammatory cytokines such as interleukin-6 (IL-6) and TNF-α [11].

Acute reaction after radiation is caused by the lack of balance between the destruction of cells due to radiation and new cells production. Under the influence of radiation or cytostatics there occurs so called direct DNA strand damage or the creation of free radicals of reactive oxygen species (ROS), which damage cells, tissues or blood vessels. It leads to the activation of the nuclear factor kappa-light-chain-enhancer of activated B cells (NF-kB) and indirectly to increased transcription of genes for proinflammatory cytokines: IL-6, IL-1B and TNF-α, which, in turn, induce and intensify the inflammation, apoptosis and tissue damage caused by RCTH. In the next phase of reaction, so called signal-amplification stage, the cell damage is intensified in result of the activation of ceramide and caspase pathway by TNF-α and, simultaneously, there is an increased synthesis of TNF-α caused by a feedback loop again activating NK-kB. In the next stage, with accumulation of radiation dose, there occurs massive damage of mucosal integrity, defects of mucosa and submucosa (ulceration phase), which leads to bacterial and fungal infections. The damaged tissues constitute another source of production of proinflammatory cytokines including TNF-α [5].

The association between the level of TNF-α and the intensity of reaction was evaluated in a few studies [14, 15]. However, the obtained results were inconclusive. Significant increase of the TNF-α level in the serum of patients irradiated due to HNC was demonstrated in a study by Haddad et al. [16]. It was additionally relationship with the intensity of OM [16]. On the other hand, in a prospective study by Meitovitz et al. [17] a reverse association was shown - decreased level of TNF-α in the serum of irradiated HNC patients and lack of relationship between the level of TNF-α and OM intensity. The role of TNF-α in the development of OM in HNC patients was also indirectly confirmed in the studies of chemical substances used in OM therapy. Animal models showed that benzydamine and IL-11 administered to irradiated animals with HNC cause a decrease in the level of TNF-α, modification of tissue response to radiation and decrease in OM intensity [18–20].

The increase of the level of TNF-α and other proinflammatory cytokines observed in the development of OM, even after considering the known OM risk factors, does not always associate with the occurrence of OM. In clinical practice, currently it is not possible to evaluate the risk of OM in patients irradiated due to HNC and thus to select a group of patients in whom there is a possibility of radiation dose escalation without risking healthy tissue damage. The significant heterogeneity of the reaction of healthy tissues adjacent to the tumor to ionizing radiation observed in various patients may result from genetic predispositions including SNP characteristics.

So far, few studies results have been published concerning the correlation between genetic varieties and reaction of normal tissues to radiation and their radiosensitivity and these studies focused on polymorphisms of genes responsible for apoptosis, DNA repair and for protection against ROS activity [10, 21, 22].

Alsbeih et al. [21] in the analysis of data from 30 patients with nasopharyngeal cancer demonstrated an association between SNPs of genes: TGFB1 and XRCC1a (C allele of gene TGFB1 [T869C codon 10 Leu/Pro, rs1982073], A allele of XRCC1 gene [G28152A, codon 399 Arg/Gln, rs25487]) and the intensity of late skin reaction after radiations in patients with HNC. In a study involving 88 patients with HNC, Werbrouck et al. [10] showed that SNP variations of DNA repair genes: XRCC3 c.722CT/TT and Ku70c.-1310CG/GG was connected with the intensity of dysphagia in patients undergoing radiotherapy due to HNC.

In a meta-analysis of 17 studies including 656 patients and 2193 controls, a significant association was demonstrated between a wild type variant of XRCC3 (c.722C > T, p.Thr241Met, rs861539) polymorphism and early reaction after radiations (OR =1.99, 95%CI: 1.31–3.01, *P* = 0.001) among patients undergoing therapy due to various cancers. In case of HNC patients, this polymorphism significantly increased the risk of radiation complications in the head and neck area (OR =2.41, 95%CI: 1.49–3.89, *p* = 0.0003) [22].

While a study by Kornguth et al. [23] proved that faster healing of reaction after radiation in the head and neck area was characteristic for patients with C allele of gene ERCC4 T2505C polymorphism. In an analysis by Pratesi et al. [9] involving data of 101 patients with squamous cell carcinoma HNC, intense dysphagia and acute mucosal reaction after radiation occurred more frequently in patients with polymorphisms of genes XRCC1 c.1196A > G (allele A) and RAD51 c.-3429 G > C (allele C). In a study involving 188 patients with nasopharyngeal cancer the intensity of acute reaction occurred significantly more frequently in patients with one allelic variation of Wnt/β-catenin GSK3β (rs375557) gene and recessive variant of adenomatous polyposis coli gene (APC, rs454886) polymorphisms [24].

The evaluation of TNF-α as a predictive factor in the development of OM is difficult due to multi-directional activity of this cytokine depending on the type of a stimulated receptor and activated signal pathway, simultaneously leading to apoptosis and proliferation. The role of TNF-α in the pathogenesis of OM is also unclear due to the fact that TNF-α can be produced both by tumor tissues and in reaction to tissue damaging factors.

Thus, it cannot be ruled out that biochemical processes leading to the development and intensification of OM in patients undergoing RTH due to HNC do not result from the disturbance of TNF-α but of its receptor TNFR1 responsible for the apoptotic pathway.

Thus, in our study, we evaluated SNP (rs4149570, c.-1187 T > G) of the gene for TNFR1-TNFRSF1A and its relationship with the risk of acute reaction after radiation in patients irradiated due to HNC. According to our knowledge it is the first such analysis.

In our study group, OM occurred in all patients. Its intensity was increasing gradually from week 2 and 3 of RTH. It is typical for the reaction of healthy tissues to ionizing radiation, where the symptoms of reaction after radiation become apparent after application of 10–20 Gy (1–2 weeks of radiation).

Classical factors influencing the reaction after radiation in the head and neck area are mainly the radiation technique and chemotherapy, age, smoking, alcohol consumption and comorbidities [7, 25]. Our study group was quite homogeneous in terms of the treatment method. All patients were irradiated using IMRT technique. In all patients, there were similar: irradiated tissue volumes (tumor or post-operative site and regional lymphatic nodes), total doses: 60-66Gy in adjuvant treatment and 70Gy in alone radiotherapy, with fractioning of 2Gy a day. All patients completed radiotherapy and received total planned dose. Thus, we have not analyzed the factors associated with radiation technique in our report.

Our patients were treated with RCTH, the PF scheme was used 1 to 4 courses of chemotherapy were administered. Using combined radiochemotherapy resulted in significantly more frequent occurrence of OM grade 3 compared to grade 1 and 2 in Week 5 and 7 of RTH.

All patients were in similar age of 60 years old (+/− 11), majority were male. No statistically significant differences in the time of occurrence of reaction and its intensity were observed in relation to age and gender.

No patient had diabetes or collagen vascular disease. Out of the remaining factors, smoking did not affect the course of OM. However, in the 7th week of RTH grade 3 OM occurred significantly more frequently compared to grade 1 and 2 in patients with excessive alcohol consumption.

Our study was the first one to evaluate the presence of polymorphisms in TNFRSF1A gene in the context of its influence on the occurrence and intensity of OM. We demonstrated that the distribution of genotypes did not depend on the studied demographic factors. However, we found significant relationship between respective genotypes and the risk of higher OM intensity. Our study demonstrated that intensified symptoms of reaction after radiation in head and neck area occurred significantly more frequently in allele T carriers.

The degree of intensity of acute reaction depends mainly on the extent of proliferative cell loss compared to their initial number and reproduction of stem cells [26]. The observed correlations can be potentially associated with increased damage and cell loss, which, in turn, can be caused by apoptotic pathway disturbance through TNF-α receptor dysfunction.

The presence of allele T was not connected with earlier occurrence of OM symptoms. The time of occurrence of acute reaction depends on the mature cells lifetime. Thus, in case of mucous membranes of cells in head and neck area, the time of cellular turnover is short, lasting for several days, and the acute reaction occurs quickly [26]. It confirms our observations that the disturbance of TNFR1 function cannot affect the time of occurrence of the reaction.

## Conclusions

Despite many limitations resulting mainly from the study group size, our study proved that acute reaction after radiation in form of OM occurred significantly more frequently in patients who were carriers of allele T of TNFRSF1A gene. The results of our analysis require further studies, however, they suggest that in the pathomechanism of development of acute reaction after radiation within the mucous membranes of patient irradiated due to HNC, significant role may be played by TNF-α receptor and genetic alterations located in its regulating region (e.g. SNPs) may modulate the risk of occurrence of OM in patients treated with RTH.
